# Effects of Salt Concentration on a Magnetic Nanoparticle-Based Aggregation Assay with a Tunable Dynamic Range

**DOI:** 10.3390/s24196241

**Published:** 2024-09-26

**Authors:** Gabrielle Moss, Christian Knopke, Solomon G. Diamond

**Affiliations:** 1 Thayer School of Engineering, Dartmouth College, Hanover, NH 03755, USA; solomon.g.diamond@dartmouth.edu; 2 Lodestone Biomedical LLC, Lebanon, NH 03766, USA; ck@lodestonebiomed.com

**Keywords:** aggregation assay, magnetic particle spectroscopy, biosensor, tunable dynamic range

## Abstract

Magnetic nanoparticles (MNPs) can be functionalized with antibodies to give them an affinity for a biomarker of interest. Functionalized MNPs (fMNPs) cluster in the presence of a multivalent target, causing a change in their magnetization. Target concentration can be proportional to the 3rd harmonic phase of the fMNP magnetization signal. fMNP clustering can also be induced with salt. Generally, salt can alter the stability of charge stabilized fMNPs causing a change in magnetization that is not proportional to the target concentration. We have developed a model system consisting of biotinylated MNPs (biotin-MNPs) that target streptavidin to study the effects of salt concentration on fMNP-based biosensing in simulated in vivo conditions. We have found that biotin-MNP streptavidin targeting was independent of salt concentration for 0.005x to 1.00x phosphate buffered saline (PBS) solutions. Additionally, we show that our biosensor’s measurable concentration range (dynamic range) can be tuned with biotin density. Our results can be leveraged to design an in vivo nanoparticle (NP)-based biosensor with enhanced efficacy in the event of varying salt concentrations.

## 1. Introduction

Functionalized magnetic nanoparticle (fMNP)-protein binding events, transduced via magnetic spectroscopy, enable biomolecule detection within a sample solution [1,2,3]. Biomolecule binding increases fMNP hydrodynamic diameter ($D_{Hyd}$) [4,5] and/or induces the formation of fMNP aggregates [6]. The fMNP magnetic moment will have a delayed response to an external magnetic field due to $D_{Hyd}$ increases and aggregate formation. The magnetic moment response can be modeled via Brownian and Néel relaxation [7,8,9]. fMNPs have been engineered to sense biomarkers, including SARS-CoV-2 [10] and H1N1 nucleoprotein [11], in vitro. Salt induced changes in fMNP aggregation pose an obstacle to the clinical translation of fMNP-based aggregation assays for in vivo applications. Additional factors impeding aggregation assay in vivo applications include, protein corona formation [12,13] and non-specific bio-distribution. Changes in salt concentration can alter nanoparticle (NP) stability [4,14]. Which can cause changes in fMNP aggregation that are not proportional to the target biomolecule concentration. To this end, we have investigated the effects of salt concentration, in the biological range, on target induced fMNP aggregation. Our aggregation assay is a model system consisting of biotinylated MNPs (biotin-MNPs) that target streptavidin. We used Lodestone Biomedical’s Nanoparticle Characterization System (an AC magnetic spectrometer, NCS) [15] to transduce biotin-MNP-streptavidin binding into a measurable signal. The NCS emits a 1 kHz AC magnetic field with 20 mT field strength and conveys the biotin-MNP harmonic response at 3 kHz [15]. We used the phase of the 3rd harmonic ($\phi_{3}$) to assess the level of biotin-MNP aggregation [16,17] and the magnitude of the 3rd harmonic ($A_{3}$) to determine the Fe concentration present in our samples after biotinylation [18]. We studied changes in streptavidin induced biotin-MNP aggregation in phosphate buffered saline (PBS) solutions. 1x PBS is a water-based solution with an approximate pH of 7.4 and salt contents that match those found in the human body. 1x PBS contains 137 mM NaCl, 10 mM phosphate, and 2.7 mM KCl. To alter the ionic strength of PBS we diluted it with deionized water (DI), resulting in the following PBS solutions: 1.00x, 0.70x, 0.50x, 0.30x, 0.05x, 0.045x, 0.04x, 0.035x, 0.025x, 0.02x, 0.015x, 0.01x, 0.005, and 0.00x (DI). We determined biotin-MNP zeta potential and $D_{Hyd}$ in each PBS solution. Additionally, we investigated differences in biosensor response characteristics in the 1.00x, 0.50x, 0.03x, 0.015x, 0.005x, and 0.00x PBS solutions. Here, we monitored changes in biotin-MNP harmonic response when in the presence of varying amounts of streptavidin. We refer to biotin-MNP $\phi_{3}$ as a function of streptavidin concentration as a streptavidin-response curve. We compared the slope, maximum $\Delta \phi_{3}$, and linear range of each streptavidin-response curve prepared in the 1.00x, 0.50x, 0.03x, 0.015x, 0.005x, and 0.00x PBS solutions.

According to the extended Derjaguin-Landau-Verwey-Overbeek (xDLVO) theory, biotin-MNP stability will be dependent on the sum of the repulsive electrostatic double layer (EDL) force, which is dependent on biotin-MNP surface charge density and the electrolyte concentration of the diluent, and the attractive van der Waals and dipolar forces acting upon similarly charged biotin-MNPs [8,19]. A positive sum suggests stability, whereas a negative sum suggests instability and hence aggregation. If a nanoparticle (NP) has a high surface charge density and/or is in a low salt environment, particle interactions are repulsive and dominated by the EDL force [20]. On the other hand, if a NP has a low surface charge density and/or is in a high salt environment, particle interactions are attractive and dominated by the van der Waals force [20]. NP zeta potential absolute value tends to decrease in high salt environments due to a counter ion layer that forms on the NP surface, decreasing its effective surface potential and leading to instability [14]. Unstable NPs tend to aggregate, leading to an increase in NP $D_{Hyd}$. Jans et al., 2009 demonstrated the effects of electrolyte concentration on gold NP (AuNP) stability [4]. They found that the $D_{Hyd}$ of citrate capped AuNPs increased with increasing NaCl concentration. Interestingly, larger (∼100 nm) AuNPs were less stable in high salt environments compared to smaller (∼40 nm) AuNPs. MNPs are more apt to cluster, compared to NPs with non-magnetic cores, due to the attractive dipolar force acting amongst MNPs, in addition to the attractive van der Waals force [21]. Nikam et al., 2014 demonstrated the effects of electrolyte concentration and zeta potential on cobalt zinc ferrite (CZF) MNP stability [14]. Their PEGylated CZF MNPs were stable in PBS for 2 weeks, although they underwent an approximate 75 nm hydrodynamic increase in double distilled water (DDW) for the same duration, indicating instability/aggregation in DDW. Moreover, they found a linear relationship between NaCl concentration and CZF MNP zeta potential. An increase in NaCl concentration led to a decrease in zeta potential absolute value. Zeta potential absolute value decreases with increasing electrolyte concentration given an increase in EDL thickness due to surface charge screening. Given previous work investigating salt induced changes in fMNP stability, we can expect a decrease in streptavidin induced biotin-MNP aggregation with decreasing salt concentration.

In this work we also show that receptor density can be used to tune the linear region of the streptavidin-response curve for our aggregation assay. The linear region of the streptavidin-response curve is commonly referred to as the dynamic range, which is the measurable concentration range of a biosensor [22]. Takae et al., 2005 demonstrated the tunability of AuNP-based aggregation assay dynamic range via ligand density [23]. They investigated lactose functionalized AuNPs that target Ricinus communis agglutin (RCA120) lectin. Lactose-AuNP’s with 40% and 65% surface area lactose coverage had dynamic ranges spanning from 25 to 200 μg/mL and 50 to 400 μg/mL, respectively. A decrease in surface area lactose coverage led to an increase in assay sensitivity. Researchers have engineered biosensors targeting metal ions [24], small molecules [25], and proteins [26] with tunable dynamic ranges. Li et al., 2013 engineered a non-cross linking AuNP-based aggregation assay with a tunable dynamic range [27]. Their target protein, alkaline phosphatase (ALP), altered the aggregation levels of AuNPs diluted in a Tris-HCL buffer solution containing adenosine triphosphate (ATP). The dynamic range of their AuNP-based aggregation assay was tuned via the addition of metal ions to the sensing solution.

Concerning fMNP ligand density modulation, Elias et al., 2013 demonstrated a non-linear relationship between superparamagnetic MNP ligand density and cell targeting [28]. They found that optimal cell targeting occurred when the MNPs had intermediate ligand densities. This relationship was held regardless of MNP core diameter or ligand type. Generally, MNPs with 1.7 ligands per 100 nm^2^ exhibited optimal cell targeting. This topic is expanded upon in Alkilany et al., 2019 review [29] of the relationship between a NP’s (including MNPs, AuNPs, and quantum dots) ligand density and its cellular uptake. The relationship between MNP ligand density and protein sensing dynamics, transduced via MPS, were reported by Wu et al., 2021. They functionalized MNPs with polyclonal antibodies (pAbs), to lend the MNPs and affinity for SARs-CoV-2 spike or nucleocapsid proteins and monitored odd harmonic amplitudes of magnetization as the pAb-MNPs were exposed to varying concentrations of their target [30]. MNPs were functionalized with 1, 2, 3, or 4 pAbs per MNP. For pAb-MNPs targeting spike protein, a pAb:MNP ratio of 3:1 yielded a linear $A_{3}$ response from 0 to 12.5 nM spike protein. Additionally, for pAb-MNPs targeting nucleocapsid protein, a pAb:MNP ratio of 4:1 yielded a linear $A_{3}$ response from 3.13 to 400 nM nucleocapsid protein. pAb-MNPs with 1:1, 2:1, or 3:1 pAb:MNP ratios did not yield linear $A_{3}$ responses with respect to target concentration, regardless of target protein (splike or nucleocapsid). Our work differs from that of Wu et al., 2021 in that we are studying changes in biotin-MNP biosensor response characteristics (the dynamic range, slope and $\Delta \phi_{3}$), via investigating changes in biotin-MNP $\phi_{3}$ as a function of target concentration, instead of odd harmonic amplitudes. Given previous work investigating NP dynamic range tunability with ligand density, we expect an increase in biotin-MNP sensitivity with decreasing biotin density.

In this work, we verified the feasibility of using the xDLVO theory to predict fMNP target response for given salt concentrations. The results showed that biotin-MNP streptavidin targeting was independent of salt concentration for 0.005x to 1.00x PBS solutions. Additionally, we show that the dynamic range (or measurable streptavidin concentration range) for biotin-MNPs decreases with decreasing biotin density. Moreover, our biotinylation protocol and subsequent biotin-MNP physical characterization suggests that $D_{Hyd}$ and zeta potential measurements may not be reliable metrics for functionalization confirmation protocols, given that we found a non-linear relationship amongst biotin density and $D_{Hyd}$ as well as zeta potential.

## 2. Materials and Methods

### 2.1. Materials

Amine functionalized 25 nm magnetite (SHA 25) particles were purchased from Ocean Nanotech, San Diego, CA, USA. N-hydroxysuccinide (NHS)-$PEG_{4}$-biotin was purchased from Thermo Fisher Scientific, Waltham, MA, USA. Streptavidin was purchased from Rockland Immunochemicals, Pottstown, PA, USA.

### 2.2. Physical Characterization of SHA 25 MNPs

A carbon film mesh grid was dipped into a 0.5 mg Fe/mL SHA 25 solution. The SHA 25 MNPs adsorbed to the carbon film mesh grid were imaged with a combined focused ion beam scanning electron microscope (FIB-SEM, Helios 5 CX DualBeam for Materials Science, Thermo Fisher Scientific), to verify their size and shape distribution. The FIB-SEM had a current and voltage of 21 pA and 20 kV, respectively. The FIB-SEM images were analyzed with ImageJ (Fiji). ImageJ has an ‘Analyze Particles’ feature that measures the area of non-interacting particles. The ‘Analyze Particles’ feature is compatible with 8-bit binary images. We used minimum error thresholding to algorithmically threshold our image. The output of the thresholding operation was a binary image. The ‘Analyze Particles’ feature was applied to the 8-bit binary image to calculate the area of 371 SHA 25 MNPs. We algebraically manipulated the reported areas to calculate the SHA 25 MNP mean diameter and standard deviation. We conducted $D_{Hyd}$ measurements of 10 mg Fe/L SHA 25 MNP samples. The SHA 25 MNP solution was diluted with PBS. The $D_{Hyd}$ measurements were conducted via dynamic light scattering (DLS) with a Zetasizer Ultra Red (Malvern Panalytical). 1 mL of each SHA 25 solution was transferred to a disposable 10 × 10 plastic cell (DTS0012). The $D_{Hyd}$ measurements were conducted in quintuplicate at 25 °C with a light scattering collection angle of 174.7° (back scatter). To verify the zeta potential of the SHA 25 MNPs in PBS, 1 mL of a 10 mg Fe/L SHA 25 MNP solution was injected into a disposable folded capillary cell (DTS1070). The zeta potential measurements were conducted in quintuplicate, with $D_{Hyd}$ measurements before and after each zeta potential measurement to monitor MNP stability.

### 2.3. Biotinylation of SHA 25 MNPs

Two Eppendorf tubes containing 300 μL of 5 mg Fe/mL SHA 25 MNP solutions were centrifuged at 12,000 RPM for 30 min at 25 °C. Once the centrifugation was complete, the diluent was aspirated and the SHA 25 MNPs were resuspended in 300 μL of PBS. Quantities of 2 mg of NHS-$PEG_{4}$-biotin are stored in each vial of the Thermo ScientificTM No-WeighTM Format. To prevent moisture condensation, we allowed the NHS-$PEG_{4}$-biotin to equilibrate to room temperature before opening the vial. To produce biotin-$PEG_{4}$-SHA 25 MNPs with ∼10 available biotins for streptavidin binding per MNP (10 biotin-MNPs), the NHS-$PEG_{4}$-biotin was diluted to 0.50 mg/mL with DI, then 1.54 μL of the 0.50 mg/mL NHS-$PEG_{4}$-biotin solution was added to one of the Eppendorf tubes containing the SHA 25 MNP solution. To produce biotin-$PEG_{4}$-SHA 25 MNPs with ∼90 available biotins for streptavidin binding per MNP (90 biotin-MNPs), the NHS-$PEG_{4}$-biotin was diluted to 2.86 mg/mL, then 158.80 μL of the 2.86 mg/mL NHS-$PEG_{4}$-biotin solution was added to the SHA 25 MNP solution. Next, both mixtures were left to incubate at room temperature for 30 min. Once the SHA 25 MNPs were biotinylated, free biotin was removed from the solution via centrifugation. Each mixture was centrifuged at 12,000 RPM for 30 min at 25 °C 8 times. After each centrifugation, the diluent was aspirated and the 10 biotin-MNP and 90 biotin-MNP solutions were resuspended in 300 μL of DI. The $A_{3}$ of SHA 25 serial dilutions (0, 1, 2, 3, 4 and 5 g/L Fe concentrations) were measured with the NCS. The $A_{3}$ of the 10 biotin-MNP and 90 biotin-MNP solutions were compared with those of the SHA 25 MNP serial dilutions to determine the Fe concentration of the biotin-MNP solutions. Lastly, the 10 biotin-MNP and 90 biotin-MNP solutions were diluted to 1 mg Fe/mL based on their $A_{3}$.

### 2.4. Physical Characterization of Biotin-MNPs

Biotin-MNP aliquots (10 mg Fe/L concentration) were submitted to DLS, to verify their $D_{Hyd}$ and zeta potential in the 1.00x to 0.00x PBS series. The DLS measurements were conducted in quintuplicate. The zeta potential measurements were conducted in quintuplicate as well, with $D_{Hyd}$ measurements before and after each zeta potential measurement to monitor biotin-MNP stability. We measured the time evolution of biotin-MNP $D_{Hyd}$ in 1.00x PBS at room temperature for 8 h, with $D_{Hyd}$ measurements approximately every 20 min. The measurement parameters for the biotin-MNP samples were identical to those used for the physical characterization of the SHA 25 MNPs (Section 2.2).

### 2.5. Magnetic Particle Spectroscopy Measurements

To obtain streptavidin-response curves for the 90 biotin-MNPs in 1.00x, 0.50x, 0.03x, 0.015x, 0.01x, 0.005x, and 0.00x PBS solutions, streptavidin serial dilutions spanning from 0.0 μM to 2694.8 μM streptavidin were prepared in each PBS solution. Each MPS sample contained 2.5 μL of a 1 mg Fe/mL 90 biotin-MNP solution mixed with 2.0 μL of a streptavidin serial dilution sample. The 1.00x PBS 0.0 μM streptavidin sample was measured repeatedly at room temperature for 12 h, with measurements approximately every 20 min, to study the time evolution of biotin-MNP stability in 1.00x PBS. Additionally, streptavidin-response curves for the 10 biotin-MNPs were obtained in 1.00x PBS and DI. To obtain the streptavidin-response curve in 1.00x PBS for the 10 biotin-MNPs, a streptavidin serial dilution spanning from 0 μM to 193 μM streptavidin was prepared in 1.00x PBS. To obtain the streptavidin-response curve in DI for the 10 biotin-MNPs, a streptavidin serial dilution spanning from 0 μM to 322 μM streptavidin was prepared in DI. Each MPS sample was prepared in triplicate. The $\phi_{3}$ of each sample was measured in triplicate with the NCS after an approximate 40 min incubation at room temperature. At the onset of each measurement, the NCS identifies the magnetic center of each sample. Next, the sample $\phi_{3}$ is measured at 7 points along the z-axis of the sample (from bottom to top). Where the center point is located at the magnetic center of the sample. The 7 point $\phi_{3}$ measurement is repeated 4 times during each NCS measurement.

We compared the 90 biotin-MNP streptavidin-response curves in 1.00x, 0.50x, 0.03x, 0.015x, 0.01x, 0.005x, and 0.00x PBS solutions to determine the effects of salt concentration on streptavidin induced biotin-MNP aggregation. We compared the 90 biotin-MNP and 10 biotin-MNP streptavidin response curves in 1.00x PBS to determine the effects of biotin density on streptavidin induced biotin-MNP aggregation. We compared the slope, maximum $\Delta \phi_{3}$, and linear range of each streptavidin-response curve. We used analysis of covariance (ANCOVA) to determine whether the slopes of each of the curves was statistically significantly different. Specifically, Matlab’s aoctool command was used to perform the ANCOVA testing.

### 2.6. xDLVO Interparticle Energies

We modeled the repulsive EDL energy as well as the attractive van der Waals and dipolar energies acting amongst biotin-MNPs to gain further insights on particle stability in each PBS solution. The EDL energy between 2 NPs (sphere-sphere geometry) was calculated as [31,32]

$$E_{edl}=2\pi \epsilon_{0}\epsilon_{r}r_{p}\psi_{p}^{2}ln\left[1+-\kappa s\right],$$

where $\epsilon_{0}$ is the permittivity of free space, $\epsilon_{r}$ is the relative permittivity of the medium, $r_{p}$ is the biotin-MNP magnetic core radius, $\psi_{p}$ is the biotin-MNP surface potential, and κ is the inverse Debeye length. EDL energy will decrease with increasing electrolyte concentration due to changes in biotin MNP Debeye length and surface potential. The zeta potential of the biotin MNPs in each PBS solution was used in place of the surface potential. Walker et al., 2009 [31] and Fiegel et al., 2019 [32] similarly substituted NP zeta potential for surface potential in their EDL calculations. Our highest concentrated electrolyte solution, 1.00x PBS, has the following electrolyte contents: 157.00 mM Na^+^, 142.00 mM Cl^−^, 4.45 mM K^+^, and 7.30 ${\mathrm{HPO}}_{4}^{-2}$, and 4.60 mM H_2_${\mathrm{PO}}_{4}^{-1}$. We assumed electrolyte concentration decreased linearly with decreasing PBS strength. Therefore, we assumed 0.50x PBS had 50% less electrolytes than 1.00x PBS, for example. However, we handled the EDL energy calculation for DI differently to agree with reported values of the Bjerrum length $\lambda_{B}$ in pH 7 DI at room temperature. $\lambda_{B}$ is the interparticle distance at which the EDL energy is comparable in magnitude to the thermal energy of the system [33]. $\lambda_{B}$ in pH 7 DI water at room temperature is 1 μm, which corresponded to a Debye length ($\frac{1}{\kappa }$) of 4.10 μm for our biotin-MNPs in DI. van der Waals and dipolar energies remain constant with respect to electrolyte concentration. The van der Waals energy between 2 NPs was calculated as [31]

$$E_{vdw}=\frac{-A_{102}r_{p}}{12s}\left[1+\frac{14s}{\lambda }\right],$$

where $-A_{102}$ is the Hamaker constant and λ is the characteristic wavelength of the medium, which assumed to be 100 nm [32]. van der Waals attractive energy is proportional to the particle Hamaker constant. Bergsrtom et al., 2011 determined the Hamaker constant for magnetite NPs in water was 33 zJ [34]. We utilized this Hamaker constant value for our van der Waals interparticle energy calculation. The dipolar energy between 2 MNPs was calculated as [19,31]

$$E_{m}=\frac{-8\pi \mu_{0}M_{s}^{2}r_{p}^{3}}{9{\left(\frac{s}{r_{p}}+2\right)}^{3}},$$

where $\mu_{0}$ is the permeability of free space and $M_{s}$ is the magnetic saturation. Interparticle dipolar energy is proportional to the magnetic saturation of the biotin-MNPs. Wang et al., 2021 reported that Ocean Nanotech SHA 25 MNPs had a magnetic moment per particle of 1.58 ×$10^{-15}$ emu (1.58 ×$10^{-18}{\mathrm{Am}}^{2}$) [35]. We divided this value by the biotin-MNP core volume to calculate the magnetic saturation of our biotin-MNPs and ultimately the dipolar interaction energy. We modeled dipolar energy as an attarctive interaction. Dipolar energy can be either attractive or repulsive depending on the angle between the magnetic moment and the line connecting the particle centers [36]. If the angle is $0\leq \theta \leq 54.09^{{}^{\circ}}$ then the interaction is attractive, however if the angle is $54.09^{{}^{\circ}}\leq \theta \leq 90^{{}^{\circ}}$ then the interaction is repulsive. We used two-sample Kolmogorov-Smirnov (KS) tests to determine whether the total interation energy corresponding to each PBS solution had statistically significantly variations Specifically, Matlab’s kstest2 command was used to perform the KS testing.

## 3. Results

### 3.1. Physical Characterization of SHA 25 MNPs

**Figure 1:**
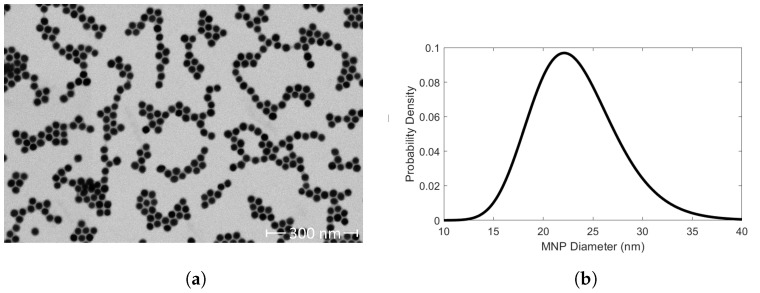
(**a**) Focused ion beam scanning electron microscope (FIB-SEM) image of Ocean Nanotech’s SHA 25 MNPs. The SHA 25 MNPs had an average diameter of 23.35 ± 4.29 nm. (**b**) Corresponding log-normal size distribution plot for the SHA 25 MNPs.

**Figure 2:**
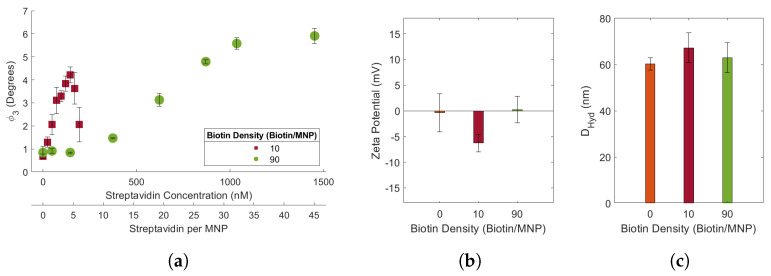
(**a**) 10 and 90 biotin-MNP $\phi_{3}$ as a function of streptavidin concentration (streptavidin-response curve) in 1.00x PBS. Where 90 biotin-MNP $\phi_{3}$ in 0.00x PBS was regarded as zero. (**b**) Zeta potential and (**c**) $D_{Hyd}$ of 10 and 90 biotin-MNPs in 1.00x PBS. The streptavidin response curve shows that the assay’s sensitivity increases as biotin density decreases.

We have determined the $D_{Hyd}$ of SHA 25 MNPs under physiologically relevant ionic conditions. SHA 25 MNPs have a 23.35 ± 4.29 nm magnetite core (Figure 1) surrounded by 4 nm organic shell composed of oleic acid and amphiphilic polymer monolayers [35]. We used 10 mg Fe/L SHA 25 MNP solutions for DLS measurements. The SHA 25 MNPs had an average $D_{Hyd}$ of 60.19 ± 1.90 nm (Figure 2c). The $D_{Hyd}$ measurement includes the 23.35 ± 4.29 nm magnetite core, the 4 nm organic layer, the amines conjugated to the particle surface, and the EDL layer surrounding the particle. We used the ‘Peak 1 Mean by Intensity Ordered by Area (nm)’ (peak 1 mean by intensity) as an indication for SHA 25 $D_{Hyd}$ instead of the z average. Z average should only be reported if the sample is monomodal, spherical, and monodisperse [37]. Some of our DLS data contained small peaks (∼2% area by intensity) on the order of a micron. We suspect these peaks are due to MNP agglomerates and/or sample contamination. Given the multimodal nature of our DLS data we have reported the peak 1 mean by intensity to indicate MNP $D_{Hyd}$, instead of the z average. We have also determined the zeta potential of SHA 25 MNPs under physiologically relevant ionic conditions i.e., in 1.00x PBS. We used 10 mg Fe/L SHA 25 MNP solutions for zeta potential measurements, which had an average zeta potential of −0.39 ± 2.98 (Figure 2b). The SHA 25 MNPs were stable during the 1st 4 zeta potential measurements. A 490.90 nm SHA 25 MNP cluster formed after the 5th zeta potential measurement. For zeta potential measurements 1 through 4, there were size distributions by intensity with average values between 58.89 and 66.30 nm. Despite their near neutral zeta potential, the SHA 25 MNPs were stable in 1.00x PBS. This suggests SHA 25 MNPs may be sterically stabilized.

### 3.2. Effects of Biotin Density on Streptavidin Induced Biotin-MNP Aggregation

Streptavidin induced biotin-MNP aggregation is biotin density dependent (Figure 2a). Biotin density is the average number of biotin conjugated to each MNP. We estimate that MNP biotin density is 2 times the streptavidin:biotin-MNP ratio at which the maximum $\phi_{3}$ occurs. The biotin-MNP receptor densities were 9.62 (∼10) and 90.08 (∼90) biotins/MNP. This assumption likely underestimates the number of biotins conjugated to each biotin-MNP. Each conjugated biotin may not be participating in streptavidin binding, due to factors including steric hinderance. It is important to note that this assumption is solely for naming convention and did not contribute to comparative analysis or our primary conclusion. Our biotinylation protocols in combination with biotin-MNP streptavidin response indicate the 90 biotin-MNPs have significantly more biotin conjugated to their surface compared to the 10 biotin-MNPs.

Our results suggest biotin-MNP streptavidin sensitivity increases with decreasing biotin density. The 10 and 90 biotin-MNPs had streptavidin-response curves with linear regions spanning from 0 to 145 nM and 145 to 1036 nM streptavidin, respectively. An inverse proportionality between ligand density and aggregation assay sensitivity was also demonstrated by Takae et al., 2004. We performed linear regressions on the 10 and 90 biotin-MNP streptavidin-response curves using the aoctool Matlab command. The linear region of the 10 biotin-MNP streptavidin-response curve had a slope and maximum $\Delta \phi_{3}$ of $0.0251^{{}^{\circ}}$/nM streptavidin and $3.54^{{}^{\circ}}$, respectively. While the linear region of the 90 biotin-MNP streptavidin-response curve had a slope and maximum $\Delta \phi_{3}$ of $0.0063^{{}^{\circ}}$/nM streptavidin and $4.11^{{}^{\circ}}$, respectively. Our results suggest that an increase in biotin density leads to a decrease in the slope and an increase in maximum $\Delta \phi_{3}$ of the streptavidin-response curve. We attribute the increase in slope with decreasing biotin density, to an increase in biotin-MNP sensitivity to streptavidin. The 10 biotin-MNPs experienced a larger increase in $\phi_{3}$ for a given nM of streptavidin, compared to the 90 biotin-MNPs. We attribute the decrease in maximum $\Delta \phi_{3}$ with decreasing biotin density, to larger and more tightly packed biotin-MNP-streptavidin aggregates at larger biotin densities. This hypothesis can be further tested by exposing biotin-MNPs, with higher and lower densities, to streptavidin concentrations that elicit a maximum $\phi_{3}$ and comparing the $D_{Hyd}$ of the biotin-MNP-streptavidin aggregates.

Moreover, we found biotin density had non-linear effects on biotin-MNP $D_{Hyd}$ and zeta potential. *T*-tests were performed to determine whether there were statistically significant variations in $D_{Hyd}$ amongst the bare SHA 25 MNP, 10 biotin-MNP, and 90 biotin-MNP samples. The *t*-tests were performed with the ttest2 Matlab command, with a 95% threshold. In PBS, the 10 and 90 biotin-MNPs had $D_{Hyds}$ of 67.18 ± 6.47 nm and 62.97 ± 1.63 nm, respectively (Figure 2c). The 10 biotin-MNPs had a $D_{Hyd}$ that was statistically significantly higher than that of the 90 biotin-MNPs and the bare SHA 25 MNPs. However, the 90 biotin-MNP $D_{Hyd}$ was not statistically significantly different from that of the bare SHA 25 MNPs. The $D_{Hyd}$ data for the bare SHA 25 MNPs as well as the 10 and 90 biotin-MNPs demonstrate a non-linear relationship between biotin density and MNP $D_{Hyd}$. In PBS, the 10 and 90 biotin-MNPs had zeta potentials of −6.28 ± 1.68 mV and 0.22 ± 2.60 nm, respectively (Figure 2b). We performed *t*-tests to determine whether there were statistically significant variations in zeta potential amongst the bare SHA 25 MNP, 10 biotin-MNP, and 90 biotin-MNP samples with a 95% threshold. Bare SHA 25 MNP zeta potential was not statistically significantly different from 90 biotin-MNP zeta potential. Although, 10 biotin-MNP zeta potential was statistically significantly different from the zeta potential of both the bare SHA 25 MNPs and the 90 biotin-MNPs. Elias et al., 2012 similarly demonstrates a non-linear relationship between HER2 density and the HER2-SPION $D_{Hyd}$ as well as zeta potential [28]. On the contrary, it has been reported that an increase in ligand density causes an increase in NP $D_{Hyd}$ and diffusion coefficient [38] as well as zeta potential [39].

### 3.3. Effects of Salt Concentration on Biotin-MNP Zeta Potential and $D_{Hyd}$

**Figure 3:**
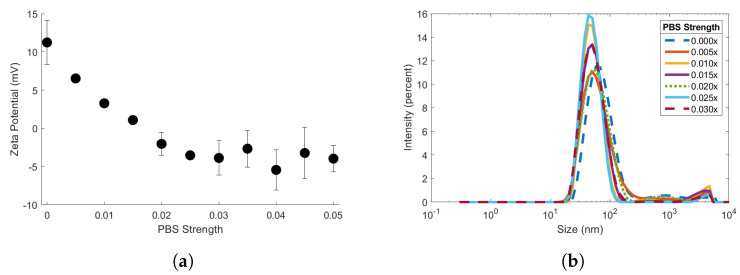
(**a**) Zeta potential of 90 biotin-MNPs in 0.05x, 0.045x, 0.04x, 0.035x, 0.03x, 0.025x, 0.02x, 0.015x, 0.01x, 0.005, and 0.000x PBS solutions. (**b**) Hydrodynamic diameter of 90 biotin-MNPs in 0.03x, 0.025x, 0.02x, 0.015x, 0.01x, 0.005, and 0.00x PBS solutions.

90 biotin-MNP zeta potential was dependent on diluent salt concentration (Figure 3a and Figure S1a). We measured the zeta potential of the 90 biotin-MNPs in the 1.00x to 0.00x PBS series. Figure 3a shows the zeta potential data for the 90 biotin-MNPs in 0.05x, 0.045x, 0.04x, 0.035x, 0.03x, 0.025x, 0.02x, 0.015x, 0.005x, and 0.00x PBS solutions. The zeta potential data for the 90 biotin-MNPs in 1.00x, 0.70x, 0.50x, and 0.30x are displayed in Figure S1a. 90 biotin-MNP zeta potential decreased linearly with increasing PBS strength from 0.00x to 0.025x PBS. In this region, zeta potential decreased from 11.24 ± 2.89 mV to −3.52 ± 0.53 mV. We performed 2-sided *t*-tests on the 90 biotin-MNP zeta potential data acquired in 0.025x, 0.035x, 0.040x, 0.045x, 0.05x, 0.30x, 0.50x, 0.70x, and 1.00x PBS solutions to determine whether the data in this region had statistically significant variations. First, we performed a Barlett Test with a 95% threshold, using the vartestn Matlab command, to determine whether the data came from normal distributions with equal variances. The null hypothesis of the Barlett Test was rejected, and we determined the zeta potential data ranging from 0.025x to 1.00x PBS came from normal distributions with unequal variances. Next, we adjusted the significance level of the *t*-test from 5% to 0.14% via Bonferonni correction to reduce the likelihood of false positive errors due to multiple comparisons. The 2-sided *t*-tests were performed with the ttest2 Matlab command. We failed to reject the null hypothesis at the correlated *p*-value of the *t*-tests and found that the zeta potential data ranging from 0.025x to 1.00x PBS came from normal distributions with equal means and unequal, but unknown, variances. Therefore, we conclude that 90 biotin-MNP zeta potential was stable in PBS solutions ranging from 0.025x to 1.00x. It is also important to note the increase in zeta potential standard deviation once diluent PBS strength surpassed 0.025x. In solutions with PBS strengths ranging from 0.005x to 0.025x the standard deviation values ranged between 0.48 to 2.04 mV. While standard deviations ranged from 2.09 to 6.62 mV in solutions with PBS strengths ranging from 0.03x to 1.00x PBS. The largest standard deviation value of 6.62 mV corresponds to the zeta potential data acquired in 0.500x PBS where the 90 biotin-MNPs had a zeta potential of 0.41 mV. It is also important to note that we observed strong zeta potential fluctuations about 0 mV. Torre et al., 2014 observed similar fluctuations about 0 mV for salt solutions ≥ 0.07 M and deemed this fraction of their data unreliable [40]. Generally, zeta potential measurements become more difficult with increasing electrolyte concentration/conductivity due to factors including Joule heating and electrode blackening.

We also measured the $D_{Hyd}$ of the 90 biotin-MNPs in the 1.00x to 0.00x PBS series (Figure 3b and Figure S1b). Figure 3b shows the size distribution plots for the 90 biotin-MNPs in 0.03x, 0.025x, 0.02x, 0.015x, 0.005x, and 0.00x PBS solutions in semilog scale. The size distribution plots for the 90 biotin-MNPs in 1.00x, 0.70x, 0.50x, 0.30x, 0.05x, 0.045x, 0.04x, and 0.035x are displayed in Figure S1b. Our data suggests that 90 biotin-MNP $D_{Hyd}$ is independent of electrolyte concentration for PBS strengths ≤1.00x. Our xDLVO theory simulation provides insights regarding biotin-MNP stability in each PBS solution. The total interparticle energy profiles corresponding to the 1.00x, 0.70x, 0.50x, 0.30x, 0.05x, 0.045x, 0.04x, 0.035x, 0.03x, 0.025x, 0.02x, 0.015x, 0.01x, and 0.005x PBS solutions were indistinguishable. For separation distances from 0 to 50 nm the total interparticle energy was negative, indicating the attractive van der Waals force was dominant amongst 90 biotin-MNPs. The total interaction energy profile corresponding to 0.00x PBS was distinct from the others. For separation distances from 0 to 22 nm the total interparticle energy was negative, indicating the attractive van der Waals force was dominant amongst 90 biotin-MNPs. Although, for separation distances from 23 to 50 nm the total interparticle energy was positive, indicating the repulsive EDL force was dominant amongst 90 biotin-MNPs.

We performed 2-sided KS tests on the total interpaticle energy data corresponding to the 1.00x, 0.70x, 0.50x, 0.30x, 0.05x, 0.045x, 0.04x, 0.035x, 0.03x, 0.025x, 0.02x, 0.015x, 0.01x, and 0.005x PBS solutions to determine whether these data had statistically significant variations. We adjusted the significance level of the KS tests from 5% to 0.05% via Bonferonni correction. We rejected the null hypothesis of the KS tests and found that the total interparticle energy data corresponding to PBS solutions ranging in strength from 0.005x to 1.000x PBS are from the same continuous distribution. Our xDLVO data suggests that 90 biotin-MNP $D_{Hyd}$ should be constant for solutions with PBS strengths ranging from 0.005x to 1.00x PBS, since 90 biotin-MNP stability is constant for these PBS solutions. Our xDLVO data also suggests the 0.00x PBS size distribution plot should be narrower with a decreased average value since the 90 biotin-MNPs are more stable in 0.00x PBS compared to solutions with PBS strengths ranging from 0.005x to 1.000x PBS. However, the size distribution plot corresponding to 0.00x PBS is not distinct from those corresponding to solutions with PBS strengths ranging from 0.005x to 1.00x PBS. In fact, 90 biotin-MNP $D_{Hyd}$ in 0.00x PBS was greater than 90 biotin-MNP $D_{Hyd}$ in 0.01x, 0.015x, 0.025x, 0.03x, 0.035x, 0.04x, 0.045x, 0.050x, 0.50x, 0.70x, and 1.00x PBS solutions. This discrepancy between xDLVO simulation data and experimental data may be due to the 4–10 nm increase in apparent NP $D_{Hyd}$ that is characteristic of $D_{Hyd}$ measurements in DI.

### 3.4. Effects of Salt Concentration on Streptavidin Induced Biotin-MNP Aggregation

**Figure 4:**
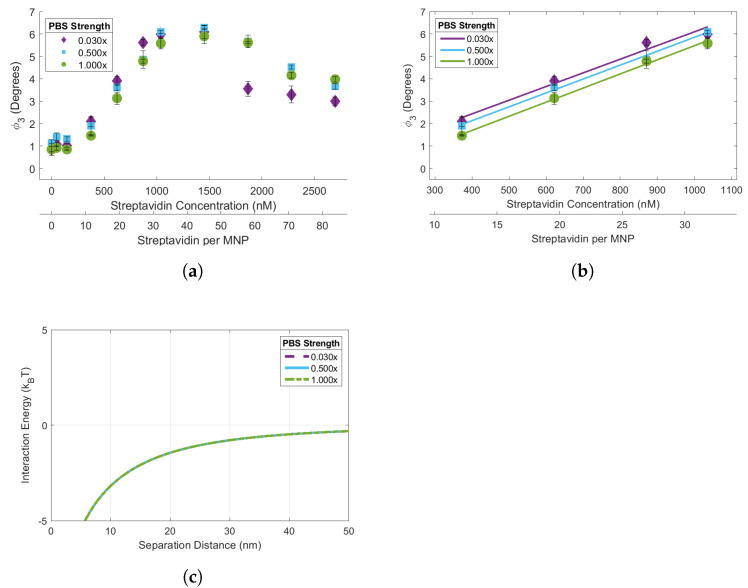
(**a**) 90 biotin-MNP $\phi_{3}$ as a function of streptavidin concentration (streptavidin-response curve) in 1.00x, 0.50x, and 0.03x PBS solutions. Where 90 biotin-MNP $\phi_{3}$ in 0.00x PBS was regarded as zero. (**b**) The linear region of each curve was fit with a linear regression, where there were no statistically significant variations amongst regression model slopes. (**c**) Total interparticle energy profiles for 90 biotin-MNPs in 1.00x, 0.50x, and 0.03x PBS solutions, where the energy profiles are indistinguishable and overlay each other. These data show that 90 biotin-MNP sensitivity to streptavidin induced aggregation was constant for 0.03x, 0.50x, and 1.00x PBS solutions. Our experimental results were supported by xDLVO simulations demonstrating that the total interparticle energy profile corresponding to each PBS solution came from the same continuous distribution.

**Figure 5:**
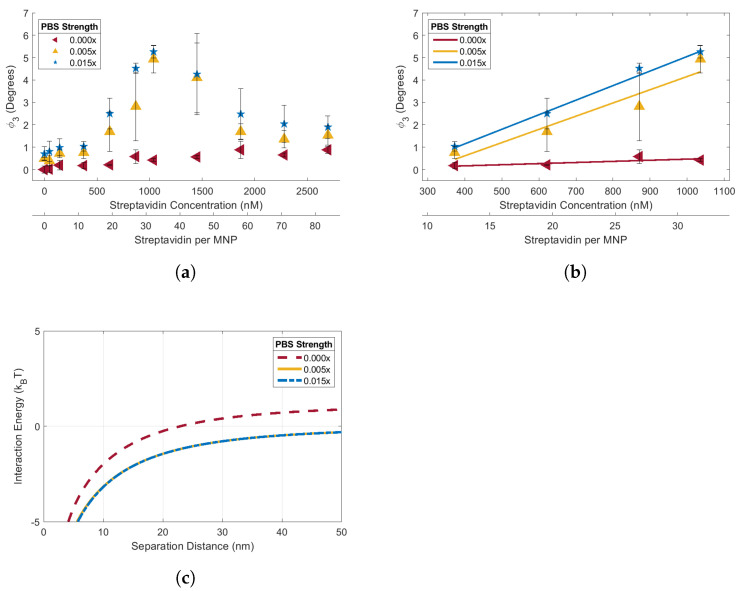
(**a**) 90 biotin-MNP $\phi_{3}$ as a function of streptavidin concentration (streptavidin-response curve) in 0.015x, 0.005x, and 0.00x PBS solutions. Where 90 biotin-MNP $\phi_{3}$ in 0.00x PBS was regarded as zero. (**b**) The linear region of each curve was fit with a regression, where the 0.00x PBS regression model slope had statistically significant variations from the regression model slopes corresponding to the 0.015x and 0.005x PBS solutions. (**c**) Total interparticle energy profiles for 90 biotin-MNPs in 0.015x, 0.005x, and 0.00x PBS solutions, where the 0.00x PBS total interparticle energy profile had statistically significant variations from the energy profiles corresponding to the 0.015x and 0.005x PBS solutions. These data show that 90 biotin−MNP senstivity to streptavidin induced aggregation was significantly decreased in 0.00x PBS. Our experimental result was supported by xDLVO simulation demonstrating that the total interparticle energy profile corresponding to the 0.00x PBS was dominated by the attractive van der Waals force for separation distances between 0 and 22 nm, while interactions were dominated by the repulsive EDL force for separation distances between 23 and 50 nm. However, total interparticle energies corresponding to the 0.015x and 0.005x PBS solutions were dominated by the attractive van der Waals force for separation distances between 0 and 50 nm.

Streptavidin-response curves for the 90 biotin-MNPs were acquired in the 1.00x, 0.50x, 0.03x, 0.015x, 0.005x, and 0.00x PBS solutions (Figure 4 and Figure 5). The streptavidin-response curves acquired in 1.00x, 0.50x and 0.03x PBS solutions demonstrate the effects of electrolyte concentration variations on streptavidin induced biotin-MNP aggregation (Figure 4). The streptavidin-response curves acquired in 0.015x, 0.005x, and 0.00x PBS solutions demonstrate the combined effects of electrolyte concentration and zeta potential variations on streptavidin induced biotin-MNP aggregation (Figure 5). Concerning the data acquired in the 1.00x, 0.50, and 0.03x PBS solutions, a decrease in electrolyte concentration increases the radius of the EDL ion cloud surrounding individual biotin-MNPs (their Debeye length, $\frac{1}{\kappa }$), causing an increase in the repulsive EDL force amongst them. The 90 biotin-MNPs had Debeye lengths of 0.2, 0.3, and 1.2 nm in 1.00x, 0.50x, and 0.03x PBS solutions, respectively. We have determined, via KS testing, that the total interparticle energy data corresponding to the 1.00x, 0.50x, and 0.03x PBS solutions are from the same continuous distribution. Therefore, 90 biotin-MNP sensitivity to streptavidin induced aggregation should be constant for 1.00x, 0.50x, and 0.03x PBS solutions, despite Debeye length increases with decreasing electrolyte concentration. The linear region for the 1.00x, 0.50x, and 0.03x PBS solutions had slopes of $0.0063^{{}^{\circ}}$/nM streptavidin, $0.0062^{{}^{\circ}}$/nM streptavidin, and $0.0061^{{}^{\circ}}$/nM streptavidin, respectively. It seems streptavidin-response curve slope underwent marginal decreases with decreasing salt concentration. To assess whether such decreases in linear region slope with decreasing salt concentration were statistically significant, we performed ANOCOVA testing on the linear region of each streptavidin-response curve. Each curve had a linear region spanning from 373.1 to 1036.4 nM streptavidin (11.58 to 32.17 streptavidin per MNP). If the biotin-MNPs are less sensitive to streptavidin induced aggregation as electrolyte concentration decreases, then we should observe a decrease in slope with decreasing electrolyte concentration. We performed our ANOCOVA test with Matlab’s aoctool command with a 95% significance threshold. We failed to reject the null hypothesis of the ANOCOVA test and found that there were not statistically significant differences amongst the slopes of the streptavidin-response curves obtained in 1.00x, 0.50x, and 0.03x PBS solutions. Therefore we conclude that the changes in EDL interparticle force, caused by electrolyte concentration variations, did not cause detectable changes in the slopes of the linear region of the streptavidin-response curves, as predicted by our xDLVO simulation. However, changes in biotin-MNP streptavidin sensitivity are detectable outside of the linear region at higher streptavidin concentrations. Each curve experiences a decrease in $\phi_{3}$ at higher streptavidin concentrations. Such decreases are due to streptavidin saturation. When there is a limited amount of streptavidin, the biotin-MNPs are forced to share, resulting in biotin-MNP aggregation. However, when in the presence of excess streptavidin, sharing is less favorable. Therefore, biotin-MNP interparticle distances increase and we observed a decrease in $\phi_{3}$ due to a decrease in dipolar interactions. The biotin-MNPs suspended in the 0.03x PBS solution experienced a greater decrease in $\phi_{3}$ at 1865.5 and 2280.2 nM streptavidin compared to those suspended in 0.50x and 1.00x PBS solutions. This greater decrease in $\phi_{3}$ in the 0.03x PBS solution may be due to greater interparticle distances amongst biotin-MNPs given the increased radius of their EDL ion cloud, and hence the greater EDL force acting amongst particles. The linear region for the 1.00x, 0.50x, and 0.03x PBS solutions had maximum $\Delta \phi_{3}$ of $4.11^{{}^{\circ}}$, $4.18^{{}^{\circ}}$, and $3.89^{{}^{\circ}}$, respectively.

Concerning the data acquired in the 0.015x, 0.005, and 0.00x PBS solutions, electrolyte concentration decreases should increase the EDL force amongst biotin-MNPs via EDL ion cloud radial increases and biotin-MNP zeta potential increases. The 90 biotin-MNPs had Debeye lengths of 1.7, 3.0, and 5170.6 nm in 0.015x, 0.005, and 0.00x PBS solutions, respectively. We have determined, via KS testing, that the total interparticle energy data corresponding to the 0.00x PBS solutions is from a different continuous distribution than those corresponding to PBS solutions ranging in strength from 0.005x to 1.00x. Therefore, we expect a change in 90 biotin-MNP sensitivity to streptavidin induced aggregation in the 0.00x PBS solution compared to the 1.00x, 0.50x, 0.03x, 0.015x, and 0.005x solutions. Specifically, 90 biotin-MNPs should be less sensitive to streptavidin induced biotin-MNP aggregation in 0.00x PBS, due to a dominant EDL force at separation distances between 23 and 50 nm. Our results show that the 90 biotin-MNPs aggregated in the presence of streptavidin when suspended in 0.015x and 0.005x PBS solutions, resulting in an increase in $\phi_{3}$ (Figure 5a). However, the 90 biotin-MNPs did not aggregate in the presence of streptavidin when suspended in 0.00x PBS (Figure 5a). The linear region for the 0.015x, 0.005x, and 0.00x PBS solutions had slopes of $0.0065^{{}^{\circ}}$/nM streptavidin, $0.0059^{{}^{\circ}}$/nM streptavidin, and $0.0005^{{}^{\circ}}/$nM streptavidin, respectively. It seems the streptavidin-response curve linear region slope decreased with decreasing electrolyte concentration (Figure 5b). We performed ANCOVA testing on the linear region spanning from 373.1 to 1036.4 nM streptavidin (11.58 to 32.17 streptavidin per MNP) to test whether the observed changes in slope are statistically significant. We rejected the null hypothesis of the ANOCOVA test with a 95% significance threshold and found that there are statistically significant differences amongst the slopes of the streptavidin-response curves obtained in 0.015x, 0.005x, and 0.00x PBS solutions. It is important to note that although the 0.015x streptavidin-response curve has a larger slope than the curve obtained in 0.005x PBS, this variation in slope is not statistically significant. Moreover, the variations in slope amongst the curves obtained in 0.005x, 0.015x, 0.03x, 0.50x, and 1.00x PBS are not statistically significantly different. The linear region for the 0.015x, 0.005x, and 0.00x PBS solutions had maximum $\Delta \phi_{3}$s of $4.22^{{}^{\circ}}$, $4.16^{{}^{\circ}}$, and $0.25^{{}^{\circ}}$, respectively. Our xDLVO simulation data was in agreement with our streptavidin-response curve data given that biotin-MNP interactions were constant in 1.00x, 0.50x, 0.03x, 0.015x, and 0.005x PBS as predicted by the indistinguishable total interparticles energies corresponding with each PBS solution. On the other hand, biotin-MNP interactions became more repulsive and less sensitive to streptavidin induced aggregation in 0.00x due to a significant increase in EDL energy amongst biotin-MNPs (Figure 5a) and a repulsive total interaction energy for separation distances between 23 and 50 nm. Therefore we conclude that streptavidin induced biotin-MNP aggregation can be predicted with xDLVO simulation of the van der Waals, EDL and dipolar interparticle energies.

## 4. Discussion

SHA 25 MNPs diluted in PBS had a $D_{Hyd}$ of 60.19 ± 1.90 nm (Figure 2c). Previous work from Wu et al., 2021, reports SHA 25 MNPs had a $D_{Hyd}$ of 28.28 ± 10.38 nm in DI after 30 min of ultrasonication [35]. Our SHA 25 MNP measurements were conducted without ultrasonication or other means of sample manipulation. It is important to note that the choice of diluent and Fe concentration can affect the accuracy of $D_{Hyd}$ measurements. DLS measurements conducted in DI typically yield $D_{Hyd}$ values 2-10 nm larger than the particle’s actual $D_{Hyd}$ [41]. This inaccuracy is attributed to the absence of ions in solution to shield long distance interparticle interactions [41]. Lim et al., 2013 reported an approximate 148 nm increase in MNP $D_{Hyd}$ due to an increase in the sample solution’s Fe concentration from 100 to 250 mg Fe/L for 18 nm superparamagnetic MNPs [37]. Constant $D_{Hyd}$ data was acquired for the 18 nm superparamagnetic MNPs at Fe concentrations between 10 and 50 mg Fe/L. Therefore, we conducted our $D_{Hyd}$ measurements with 10 mg Fe/L MNP samples for our 23.35 ± 4.29 nm core MNPs. Moreover, MNP core size must also be considered when determining an optimal Fe concentration for $D_{Hyd}$ measurements. At a constant Fe concentration, larger MNPs have a lower collision frequency, hence their $D_{Hyd}$ would more accurately reflect the physical properties of individual MNPs, compared to smaller MNPs. When comparing literature values of NP $D_{Hyds}$ one must consider the diluent, NP concentration, and core size to make an informed comparative analysis.

After biotinylating SHA 25 MNPs with ∼10 and 90 biotins/MNPs, we investigated the effects of ligand density on streptavidin induced biotin-MNP aggregation. We estimate that MNP biotin density is 2 times the streptavidin:biotin-MNP ratio at which the maximum $\phi_{3}$ occurs. Traditional methods for NP ligand density quantification [38] include nuclear magnetic resonance (NMR), fluorescently labelled ligands, and absorption spectroscopy. Previous works have used an absorption spectroscopy technique, wherein the ligand density of AuNPs was estimated by the shape of the absorbance versus added protein curve [42,43]. The protein concentration at which the absorbance curve plateaued indicated the amount of ligand bound to the AuNP surface. Our biotin-MNP ligand density quantification method is similar, however we multiply the peak value of our target response curve with 2. The multiplication factor is applied since our added protein is an aggregation inducing agent. We assume that each streptavidin is bound to 2 biotin-MNPs. Streptavidin-response curves were obtained for the 10 and 90 biotin-MNPs in 1.00x PBS. We compared the slope, maximum $\Delta \phi_{3}$, and range of the linear region of each streptavidin-response curve. Our results suggest an increase in streptavidin-response curve slope with decreasing biotin density. In other words, biotin-MNP streptavidin sensitivity increased with decreasing biotin density. Additionally, we found the streptavidin-response curve maximum $\Delta \phi_{3}$ decreased with decreasing biotin-density. We suspect lower biotin density MNPs form smaller and more loosely packed clusters with streptavidin, compared to high biotin density MNPs. Our results can be leveraged for MNP-based aggregation assay development. fMNP target sensitivity and dynamic range can be tuned with ligand density. Another element of our data to consider for aggregation assay development pertains to the physical characterization of the SHA 25, 10 biotin-MNPs, and 90 biotin-MNPs. Our results suggest a non-linear relationship amongst ligand density and MNP $D_{Hyd}$ as well as zeta potential. NP $D_{Hyd}$ and zeta potential are commonly used to assess the level/success of NP functionalization. Our results suggest that $D_{Hyd}$ and zeta potential may not indicate the level/success of functionalization given that the SHA 25 and 90 biotin-MNPs showed no significant variation in $D_{Hyd}$ or zeta potential. An alternative method to assess the level of NP functionalization is to expose the bare and potentially functionalized NPs to varying amounts of target and compare transducer outputs. If the potentially functionalized NPs aggregate in the presence of their target, while the bare NPs remain fully dispersed, one can presume the NPs were successfully functionalized. This method could also be used to determine NP receptor density, wherein the target concentration corresponding to the maximum transducer output is an approximate indication of NP receptor density.

To determine the effects of electrolyte concentration on streptavidin induced biotin-MNP aggregation, streptavidin-response curves were obtained for the 90 biotin-MNPs in 1.00x, 0.50x, 0.03x, 0.015x, 0.005x, and 0.00x PBS solutions. We compared the slope, maximum $\Delta \phi_{3}$, and range of the linear region of each streptavidin-response curve. There were no significant variations amongst linear region slope or range for the streptavidin-response curves prepared in 1.00x, 0.50x, 0.03x, 0.015x, and 0.005x PBS solutions. The biotin-MNPs formed aggregates with streptavidin in the 1.00x, 0.50x, 0.03x, 0.015x, and 0.005x PBS solutions, however the biotin-MNP were insusceptible to streptavidin induced aggregation in 0.00x PBS. The differences in biotin-MNP interparticle interactions were predicted with our xDLVO simulation. According to our simulation, interparticle interactions were dominated by the attractive van der Waals force in solutions with PBS strengths ranging from 0.005x to 1.00x PBS, while in 0.00x PBS interparticle interactions were dominated by the repulsive EDL force at particle separation distances between ∼23 nm and 50 nm. We have demonstrated that seemingly incremental changes in NP zeta potential and/or diluent electrolyte concentration can yield significant changes in NP-target interactions. For example, when suspended in 0.005x PBS (a relatively low electrolyte concentration diluent) the 90 biotin-MNPs had a zeta potential of 6.53 ± 0.72 mV and particle interactions were dominated by the attractive van der Waals force for separation distances (s) 0≤ s ≤50. However, after seemingly incremental changes in diluent to 0.00x PBS and 90 biotin-MNP zeta potential to 11.24 ± 3.88 mV, particle interactions were dominated by the repulsive EDL force for 23≤ s ≤50, resulting in no detectable levels of streptavidin induced biotin-MNP aggregation. Therefore, our study highlights the importance of xDLVO simulation during aggregation assay design to tune fMNP zeta potential to the diluent electrolyte concentration and pH for optimal target binding and colloidal stability. Concerning the effects of electrolyte concentration on the streptavidin-response curve maximum $\Delta \phi_{3}$, no correlation was found.

$D_{Hyd}$ and zeta potential measurements were obtained for the 90 biotin-MNPs in the 1.00x to 0.00x PBS series to assess the effects of electrolyte concentration on colloidal stability. Ninety biotin-MNP zeta potential decreased linearly in solutions with PBS strengths ranging from 0.025x to 0.00x PBS (Figure 3a). While 90 biotin-MNP zeta potential was constant in solutions with PBS strengths ranging from 1.00x to 0.03x PBS (Figure 3a). Ninety biotin-MNP $D_{Hyd}$ showed no trend with electrolyte concentration, therefore 90 biotin-MNP $D_{Hyd}$ is independent of electrolyte concentration in solutions with PBS strengths ≤ 1.00x. Our 90 biotin-MNP $D_{Hyd}$ data for solutions with PBS strengths < 1.00x is limited because it does not account for aggregate growth over time. Our $D_{Hyd}$ data was acquired directly after the 90 biotin-MNPs were mixed with each PBS solution, for those < 1.00x. Hence, our data reflect the initial 90 biotin-MNP size after exposure to diluents of varying PBS strength. Salt induced NP aggregates may grow in size over time depending on the salt concentration of the diluent [40,44,45,46].

## 5. Conclusions

Our work shows that (1) biotin-MNP streptavidin targeting was independent of salt concentration for 0.005x to 1.00x PBS solutions. Wherein there were no statistically significant variations amongst the slopes of the streptavidin-response curves corresponding to the 0.005x to 1.00x PBS solutions. Moreover, our data suggest that 90 biotin-MNP initial $D_{Hyd}$ is independent of electrolyte concentration in solutions with PBS strengths <1.00x. Further research is required to verify whether 90 biotin-MNP $D_{Hyd}$ changes with time in <1.00x PBS solutions. (2) The slope, maximum $\Delta \phi_{3}$, and linear range of a streptavidin-response curve can be tuned with biotin density. As biotin density decreases the streptavidin-response curve dynamic range shifts to lower concentrations, the slope increases, and the maximum $\Delta \phi_{3}$ decreases. (3) Biotin density has a non-linear effect on biotin-MNP $D_{Hyd}$ and zeta potential, therefore methods other than $D_{Hyd}$ and zeta potential measurements are required for functionalization confirmation protocols. (4) xDLVO simulations can accurately predict fMNP biosensor response characteristics. Samples with insignificant variations amongst their interparticle energies yielded streptavidin-response curves with constant slopes and dynamic ranges. Significant changes in interparticle energy yielded statistically significant changes in the slope, maximum $\Delta \phi_{3}$, and dynamic range of the streptavidin-response curve linear region. Further research is required to highlight the transition region, wherein the biotin-MNP streptavidin response curve experiences significant decreases in slope and maximum $\Delta \phi_{3}$. Our data transitions from streptavidin-response curves with constant slope and dynamic range to no aggregation. An electrolyte concentration range wherein biotin-MNP streptavidin induced aggregation is transitional can be identified with xDLVO theory simulation.

## Data Availability

The data presented in this study are available on the corresponding author’s Figshare.

## Supplementary Materials

The following supporting information can be downloaded at: https://www.mdpi.com/article/10.3390/s24196241/s1, Figure S1: (a) Zeta potential of 90 biotin-MNPs in 1.0x, 0.7x, 0.5x, and 0.3x PBS solutions. (b) Hydrodynamic diameter of 90 biotin-MNPs in 1.000x, 0.700x, 0.500x, 0.300x, 0.050x, 0.045, 0.040x, and 0.035x PBS solutions.; Figure S2: (a) 10 biotin-MNP $\phi_{3}$ as a function of streptavidin concentration (streptavidin-response curve) in 0.0x and 1.0x PBS. Where 10 biotin-MNP $\phi_{3}$ in 0.0x PBS is regarded as zero. (b) Zeta potential and (c) $D_{Hyd}$ of 10 biotin-MNPs in 0.0x and 1.0x PBS. These data show that 10 biotin-MNP sensitivity to streptavidin induced aggregation is significantly decreased in 0.0x PBS, given the increased electrostatic double layer (EDL) force amongst particles. The 10 biotin-MNPs experience a greater EDL force in 0.0x PBS, compared to 1.0x PBS, given that it’s void of salt and the 10 biotin-MNPs have a greater zeta potential.; Figure S3: Time evolution of 90 biotin-MNP (a) $\phi_{3}$ and (b) hydrodynamic diameter while suspended in 1.00x PBS at room temperature. Where 90 biotin-MNP $\phi_{3}$ in 0.00x PBS was regarded as zero. There were no statistically significant variations amongst the values for 90 biotin-MNP $\phi_{3}$ over time (99.99% significance threshold). These data suggest that 90 biotin-MNPs remain stable in 1.00x PBS at room temperature for at least 12 h.
